# Takotsubo Cardiomyopathy With a Rapidly Resolved Left Ventricular Thrombus

**DOI:** 10.1177/2324709617734238

**Published:** 2017-09-28

**Authors:** Abdel Anabtawi, Paola C. Roldan, Carlos A. Roldan

**Affiliations:** 1 University of New Mexico, Albuquerque, NM, USA; 2 New Mexico VA Health Care Center, Albuquerque, NM, USA

**Keywords:** takotsubo cardiomyopathy, left ventricular thrombus, anticoagulation, echocardiography

## Abstract

This article presents the case of a 53-year-old man who presented with acute right superficial femoral and popliteal arterial thrombosis for which he underwent an emergent uncomplicated thrombectomy. He denied preceding cardiovascular or neurologic symptomatology and had no history of coronary or peripheral arterial disease, trauma, hypercoagulability, or malignancy. However, he reported having several days of intense emotional stress prior to presentation. His cardiac exam was normal, his electrocardiogram showed normal sinus rhythm and nonspecific ST-T wave abnormalities, and his troponin levels were normal. Transthoracic echocardiography (TTE) revealed a large (2.4 × 2 cm) apical left ventricle (LV) thrombus, LV apical akinesis, and LV ejection fraction of 40% to 45%. Coronary angiography revealed only luminal irregularities. A repeat TTE performed 3 days after initiating unfractionated heparin revealed complete resolution of the LV thrombus. The patient had an uneventful clinical course and was discharged home in stable condition on oral anticoagulants. The lower incidence of LV thrombus in takotsubo cardiomyopathy (TC) of 1.3% in comparison to 4% to 8% in acute myocardial infarction due to coronary artery disease in the current era of early reperfusion may be explained by the lower extent of ischemic myocardial necrosis associated with TC. This case suggests that the lower extent of myocardial necrosis in TC may also lead to faster resolution of LV thrombus. Therefore, earlier follow-up with TTE (within 2 weeks) and shorter duration of anticoagulation (<3 months) may be considered in patients with TC complicated by LV thrombus formation with or without systemic embolism.

## Introduction

Takotsubo cardiomyopathy (TC), also known as stress cardiomyopathy or apical ballooning syndrome, is a type of cardiomyopathy in which there is a sudden temporary ischemic systolic dysfunction of the heart due to catecholamine excess, coronary vasospasm, or microvascular dysfunction.^1^ TC is far more common (80% to 100% of cases) in postmenopausal women.^2^ TC is usually, but not always, triggered by emotional stress.^2^ TC is characterized by transient apical ballooning of the left ventricle (LV) with wall motion abnormalities in the mid to distal and apical regions and is generally associated with a decrease in LV ejection fraction.^3-5^ Right ventricular (RV) involvement has been described in one third of cases.^4-6^ Spontaneous recovery of the LV and RV myocardial dysfunction is usually observed within a median of 7 days.^2,5-7^ Patients initially present with chest pain and dyspnea, mimicking an acute coronary syndrome (ACS), and therefore treatment is begun as for an ACS.^2,5,8^ Once obstructive (>50% stenosis) coronary artery disease (CAD) is excluded via coronary angiography, treatment for LV dysfunction with beta-blockers and renin-angiotensin-aldosterone inhibitors is usually employed.^2,5,7,9^

Electrocardiographic abnormalities in TC include ST-segment changes, predominantly ST-segment elevation in the precordial leads.^10^ Occasionally, QT prolongation or atrioventricular conduction abnormalities are seen.^11,12^ Initially, serum cardiac troponin levels are elevated in most patients (median troponin level is 7.7 times the upper limit of normal with interquartile range of 2.2 to 24) as reported in the International Takotsubo Registry study,^13,14^ while creatinine kinase levels are generally normal or mildly elevated (median creatinine kinase of 0.85 times the upper limit of normal with interquartile range of 0.52 to 1.48). These features make distinguishing TC from ACS a diagnostic challenge. Therefore, patients typically undergo emergent coronary angiography to exclude obstructive epicardial CAD. LV angiography and echocardiography demonstrate characteristic mid to apical ballooning of the LV with hyperdynamic basal segments.^2,5-7,14^

The overall prognosis of patients with TC is similar to that of patients with ACS. TC can be complicated by LV thrombus formation with or without systemic embolism, ventricular tachyarrhythmias, heart failure, cardiogenic shock due to significant LV systolic dysfunction, or less commonly due to obstructive cardiomyopathy-like pathophysiology caused by hypercontractile basal segments of the LV, ventricular rupture, or death.^2,5-7,11,12,15-21^

## Case Report

We present the case of a 53-year-old male patient with a history of hypertension, hyperlipidemia, and obstructive sleep apnea that presented with acute right lower extremity pain and was diagnosed with right superficial femoral artery and popliteal artery thrombosis.

He denied having any preceding chest pain or any other cardiac or neurologic symptomatology. He had no history of CAD, peripheral arterial disease, trauma, hypercoagulability, or malignancy. However, he reported having several days of intense emotional stress prior to presentation. His cardiac exam was normal and his electrocardiogram (ECG) revealed nonspecific ST-T wave abnormalities. His laboratory data and troponin levels were normal. The patient underwent an emergent uncomplicated femoral and popliteal arterial thrombectomy.

**Figure 1.**
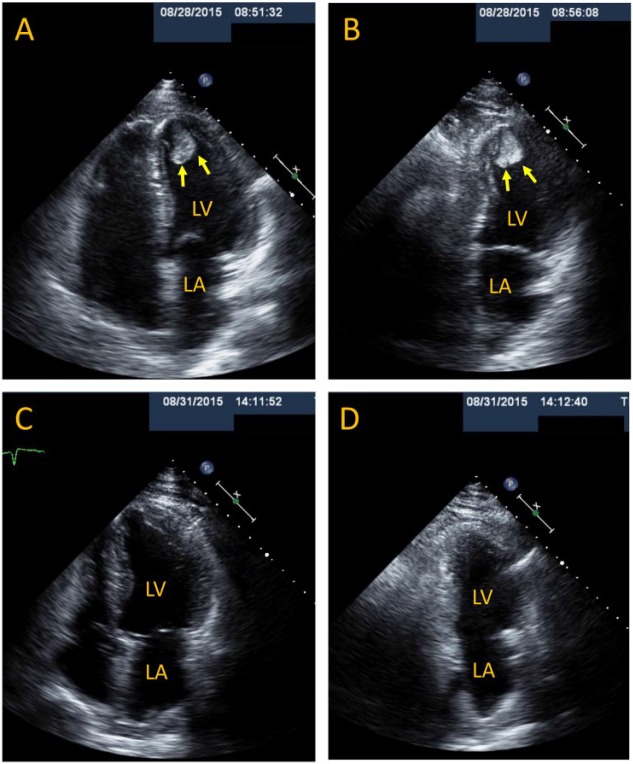
Resolution of a large apical LV thrombus within 3 days of anticoagulation. (A, B) Two-dimensional apical 4-chamber (A) and 2-chamber (B) TTE views demonstrate a large (2.4 × 2 cm), elongated, with a narrow attachment, mobile, and heterogeneously echoreflectant apical LV thrombus (arrows). Repeat 4-chamber (C) and 2-chamber (D) TTE views 3 days after initiation of intravenous unfractionated heparin demonstrate complete resolution of the apical LV thrombus. Abbreviations: LA, left atrium; LV, left ventricle; TTE, transthoracic echocardiography.

Postoperative echocardiogram on day 1 of hospitalization demonstrated LV apical akinesis, an estimated LV ejection fraction of 40% to 45%, and a large (2.4 × 2 cm), pedunculated, and mobile apical LV mass suggestive of an LV thrombus (Figure 1A and B). Cardiac magnetic resonance imaging (MRI) performed on day 2 confirmed a large apical LV thrombus and coronary angiography showed only luminal irregularities.

On day 3 of intravenous unfractionated heparin a repeat transthoracic echocardiography (TTE) to further characterize the LV thrombus with 3D imaging revealed complete resolution of the LV thrombus with persistent LV apical akinesis (Figure 1C and D). The patient had an uneventful clinical course and was discharged home on day 5 on warfarin for at least 3 months.

## Discussion

To the best of our knowledge, this is the first case of TC complicated by peripheral arterial embolism and a large apical LV thrombus that resolved completely within 3 days of intravenous unfractionated heparin.

The differential diagnosis of myocardial infarction with nonobstructive CAD includes the following: (1) TC, (2) coronary vasospasm due to Prinzmetal’s angina (more common in young women) or drug-induced vasospasm secondary to the use of cocaine or amphetamines, (3) a hypercoagulable state with in situ coronary thrombosis, (4) vasculitis with or without in situ thrombosis, (5) coronary embolism from infective or noninfective valve vegetations or atrial fibrillation, or (6) myocarditis.^2,7,10,14,22-24^ Our patient’s clinical, laboratory, and imaging data did not fit any of the last 5 clinical conditions. He denied any tobacco use, had a negative drug screen, a negative hypercoagulable state, his coronary arteries were angiographically nearly normal, had no other cardioembolic source seen on echocardiography, was in normal sinus rhythm throughout his hospital stay, and the absence of myocardial inflammation on MRI and segmental rather than global LV dysfunction on MRI and echocardiography were inconsistent with myocarditis.

The initial diagnosis of TC in our patient was challenging because of the absence of chest pain and lack of diagnostic ECG changes and no troponin elevation. However, the patient did report having several days of intense emotional stress prior to presentation. Since TC can often represent a diagnostic challenge for clinicians, specific diagnostic criteria for TC have been proposed.^25-27^ This case fulfils the revised Mayo Clinic criteria listed below.

1. Transient hypokinesis, akinesis, or dyskinesis of the LV mid segments with or without apical involvement; the regional wall motion abnormalities extend beyond a single epicardial coronary vascular distribution; a stressful trigger is often, but not always present.
2. Absence of obstructive CAD or absence on angiography of acute coronary plaque rupture.
3. New ECG abnormalities (either ST-segment elevation and/or T-wave inversion) or modest elevation in cardiac troponin enzymes.
4. Absence of pheochromocytoma or myocarditis.

Left ventricular thrombi has been identified in 1.3% of patients with TC and about 1% of patients have cardioembolic complications.^7,13-16^ Thrombus formation is more common in the LV apex, but can infrequently affect the RV. The clinical presentation of thromboembolism can occur at initial presentation or any time during the course of the disease.^2,3,5,15-17^

The pathogenesis of LV thrombus formation in TC is presumably due to the contact of blood with the endothelium-denuded endocardium and the actively inflamed and necrotic subendocardium compounded by blood stasis in the presence of mid to apical wall motion abnormalities or LV aneurysm.^15-17,28^

LV apical thrombus in TC is usually detected with noncontrasted and sometimes contrasted echocardiography.^3,5,15^ Cardiac MRI and computed tomography may also be used to identify and further characterize an LV or RV thrombus.^29,30^ However, these imaging techniques are rarely used for this purpose since the sensitivity of TTE for detection of LV thrombus is >95%.

TC complicated by thromboembolism is associated with an increase in morbidity and mortality.^15-18,21^ Therefore, anticoagulation therapy is indicated in specific subsets of patients with TC to prevent LV thrombus formation and systemic embolism.

The recommendations for anticoagulation to prevent embolization in patients with TC and LV thrombus are derived from observational studies in patients with LV thrombus after CAD-related myocardial infarction.^31-33^ In those studies, anticoagulation for a period of 3 to 6 months was associated with a reduced rate of embolization. The duration of anticoagulation may be modified based on the rate of recovery of cardiac function and resolution of the LV thrombus.

Also, limited specific data are available to guide anticoagulant therapy to prevent LV thrombus formation and thromboembolism in patients with TC. For patients without LV thrombus but with significant anteroapical segmental wall motion abnormalities (akinesis or dyskinesis) or LV apical aneurysm, and/or severe LV systolic dysfunction (ejection fraction <30%), anticoagulation with warfarin for at least 3 months, or until LV akinesis or dyskinesis has resolved is considered reasonable.^15-18,21,31-33^

In conclusion, the lower incidence of LV thrombus in TC of 1.3% in comparison to 4% to 8% in acute myocardial infarction due to CAD in the current era of early reperfusion may be explained by the lower extent of ischemic myocardial necrosis associated with TC.^2,31-33^ Also the lower extent of myocardial necrosis and rapid recovery of LV function in TC may also lead to faster resolution of LV thrombus. As illustrated in this case, the rapid resolution of the LV thrombus suggests that repeating an echocardiogram earlier (perhaps within 2 weeks) to reassess LV wall motion abnormalities and LV thrombus regression may be appropriate in patients with TC. This strategy may shorten the duration of and decrease the potential risks associated with oral anticoagulation. However, a prospective cross-sectional and longitudinal study using serial echocardiography in patients with TC complicated by LV thrombus is needed to confirm this hypothesis.
